# Frontiers of Cranial Base Surgery: Integrating Technique, Technology, and Teamwork for the Future of Neurosurgery

**DOI:** 10.3390/brainsci13101495

**Published:** 2023-10-23

**Authors:** Corneliu Toader, Lucian Eva, Catalina-Ioana Tataru, Razvan-Adrian Covache-Busuioc, Bogdan-Gabriel Bratu, David-Ioan Dumitrascu, Horia Petre Costin, Luca-Andrei Glavan, Alexandru Vlad Ciurea

**Affiliations:** 1 Department of Neurosurgery, “Carol Davila” University of Medicine and Pharmacy, 020021 Bucharest, Romania; corneliu.toader@umfcd.ro (C.T.); razvan-adrian.covache-busuioc0720@stud.umfcd.ro (R.-A.C.-B.); david-ioan.dumitrascu0720@stud.umfcd.ro (D.-I.D.); horia-petre.costin0720@stud.umfcd.ro (H.P.C.); luca-andrei.glavan0720@stud.umfcd.ro (L.-A.G.); prof.avciurea@gmail.com (A.V.C.); 2 Department of Vascular Neurosurgery, National Institute of Neurology and Neurovascular Diseases, 077160 Bucharest, Romania; 3 Department of Neurosurgery, Dunarea de Jos University, 800010 Galati, Romania; 4 Department of Neurosurgery, Clinical Emergency Hospital “Prof. Dr. Nicolae Oblu”, 700309 Iasi, Romania; 5 Department of Ophthalmology, “Carol Davila” University of Medicine and Pharmacy, 020021 Bucharest, Romania; 6 Clinical Hospital of Ophthalmological Emergencies, 010464 Bucharest, Romania; 7 Neurosurgery Department, Sanador Clinical Hospital, 010991 Bucharest, Romania

**Keywords:** cranial base surgery, minimally invasive techniques, intraoperative neuromonitoring, advanced imaging, robotics in neurosurgery, radiosurgery, gamma knife, cyberknife, interdisciplinary collaboration, functional guidance, patient-centric care, endocrinology, biomaterial science, machine learning, future of neurosurgery

## Abstract

The landscape of cranial base surgery has undergone monumental transformations over the past several decades. This article serves as a comprehensive survey, detailing both the historical and current techniques and technologies that have propelled this field into an era of unprecedented capabilities and sophistication. In the prologue, we traverse the historical evolution from rudimentary interventions to the state-of-the-art neurosurgical methodologies that define today’s practice. Subsequent sections delve into the anatomical complexities of the anterior, middle, and posterior cranial fossa, shedding light on the intricacies that dictate surgical approaches. In a section dedicated to advanced techniques and modalities, we explore cutting-edge evolutions in minimally invasive procedures, pituitary surgery, and cranial base reconstruction. Here, we highlight the seamless integration of endocrinology, biomaterial science, and engineering into neurosurgical craftsmanship. The article emphasizes the paradigm shift towards “Functionally” Guided Surgery facilitated by intraoperative neuromonitoring. We explore its historical origins, current technologies, and its invaluable role in tailoring surgical interventions across diverse pathologies. Additionally, the digital era’s contributions to cranial base surgery are examined. This includes breakthroughs in endoscopic technology, robotics, augmented reality, and the potential of machine learning and AI-assisted diagnostic and surgical planning. The discussion extends to radiosurgery and radiotherapy, focusing on the harmonization of precision and efficacy through advanced modalities such as Gamma Knife and CyberKnife. The article also evaluates newer protocols that optimize tumor control while preserving neural structures. In acknowledging the holistic nature of cranial base surgery, we advocate for an interdisciplinary approach. The ecosystem of this surgical field is presented as an amalgamation of various medical disciplines, including neurology, radiology, oncology, and rehabilitation, and is further enriched by insights from patient narratives and quality-of-life metrics. The epilogue contemplates future challenges and opportunities, pinpointing potential breakthroughs in stem cell research, regenerative medicine, and genomic tailoring. Ultimately, the article reaffirms the ethos of continuous learning, global collaboration, and patient-first principles, projecting an optimistic trajectory for the field of cranial base surgery in the coming decade.

## 1. Prologue: An Overview

### 1.1. Study Design and Methodology

The endeavor for this research was based on a comprehensively point of view regarding the evolution of multimodel treatment of skull base pathologies, current updates, and emerging computational systems. Firstly, we delved into microscopic and endoscopic assisted approaches, as well as intraoperative electrophysiological monitoring and adjuvant therapies, comparing the postoperative outcome, and long-term prognosis in different therapies. We performed an analytical exploration on Web of Science and PubMed datasets, using the following terms: “Skull Base Surgery”, “Minimally Invasive Techniques”, “Endoscopic Approach”, “Intraoperative Neuromonitoring”, “Cranioplasty”, “Biomaterials”, “Radiosurgery”,” Stereotactic surgery”, “Gamma Knife”, “CyberKnife”, “Patient-Centric Care”, “Machine Learning”, “Deep Learning”, and “Neurosurgery Future”. Our review emphasized the historical timeline of skull base treatment, showcasing the milestone discoveries.

### 1.2. Synthesizing the Historical Timeline: From Rudimentary Techniques to the Forefront of Neurosurgical Intervention

In the period antedating the ubiquitous presence of antibiotics, pioneering neurosurgeons such as Schloffer, Cushing, and Hirsch stood at the forefront of advancements in cranial surgery. Their endeavors were characterized by the exploration and development of avant-garde techniques to gain access to intracranial structures. The transnasal approach, specifically targeting the pituitary fossa, is a notable example of such pioneering work. The relatively low mortality rate of this approach, standing at an impressive 5%, was, however, marred by the omnipresent threat of meningitis as the chief cause of death. Despite its efficacy, the transnasal approach was gradually eclipsed as Cushing and his contemporaries gravitated towards the transcranial route over the ensuing one to two decades. This transition, driven by the imperative to circumvent the inherent infectious risks of the former technique, exemplifies the dynamic nature of neurosurgical techniques that evolve in tandem with emerging medical challenges and exigencies [1].

The genesis of Anterior Skull Base (ASB) surgery as a distinct field is anchored in the innovations of the 1940s. Dandy’s instrumental contributions are emblematic of this era, particularly his surgical strategy via the anterior cranial fossa for the excision of orbital tumors and his subsequent expansion of the resection to incorporate the ethmoidal regions. In a parallel trajectory, Ray and McLean championed a novel combined transorbital and transcranial method for addressing retinoblastomas. Adding to the burgeoning body of work in this domain, in 1954 a comprehensive transcranial-transfacial approach was described tailored for managing malignancies located in the paranasal sinuses and their immediate anatomical vicinities. This surgical blueprint, having gained considerable traction and endorsement during 1960’, underscored the emergence of ASB surgery as a specialized niche within the broader realms of neurosurgery and head and neck surgical disciplines. Moreover, during an exposition on craniofacial resection, meticulously crafted for the treatment of ethmoid carcinoma and inclusive of the cribriform plate resection, augmented the repertoire of ASB surgical techniques. In subsequent discourse and practice, the anterior craniofacial resection (ACFR) acquired a reputation as the quintessential intervention for ASB tumors, especially those with origins in the paranasal sinuses and encroachments into the skull base [2].

Pertaining to the lateral avenues to the skull base, the early 20th century was defined by a renaissance of surgical approaches, with the innovations and contributions of Harvey Cushing at its epicenter. Cushing’s pioneering methodologies were highlighted by the inception of the extended bilateral suboccipital technique, crafted specifically for the resection of tumors. Anchored in his conviction, he asserted that the surgical intervention should be judiciously limited to the tumor’s nucleus to safeguard the functional integrity of the cranial nerves and to obviate undue perturbations to the intricate vascular networks of the brainstem. Furthermore, Cushing, with a perspicacious insight, underscored the quintessence of adopting precision-driven and nuanced surgical modalities, judicious modulation of cerebrospinal fluid dynamics, and a minimization of cerebellar manipulation to create an optimal surgical milieu. Such foundational tenets, propounded by Cushing, have been instrumental in sculpting the landscape of present-day surgical praxis and remain deeply ingrained in the ethos of contemporary skull base surgery [3].

A subsequent landmark in cranial surgical evolution was heralded by the advent of microvascular surgery in the 1960s. Pioneered by Jacobsen and Suarez, and later honed by Nakagama and his associates, this innovative paradigm ushered in a new chapter in cranial reconstructive strategies. Central to this was the concept of free flap transplantation, which, over the decades, has burgeoned into a sine qua non for cranial base reconstructions following exenteration procedures. While the utility of free grafts and regional flaps is undeniable in the reconstitution and morphological restoration of these multifaceted cranial zones, the supremacy of free flap transplantation lies in its unparalleled capability to effectuate a definitive demarcation between the intracranial enclave and the upper alimentary and respiratory tracts [4].

Spanning the annals of medical history, surgical interventions with the skull base as the focal point have invariably found a nexus in the disciplines of neurosurgery and otolaryngology. The surgical gamut in this context is extensive, encapsulating procedures such as resection of paragangliomas with an epicenter in the skull base and consequent endocranial extension, transnasal hypophysectomies, and diverse methodologies including cranio-facial and transfrontal techniques tailored for afflictions and neoplasms of the anterior cranial vault. Moreover, the choice between fronto-temporal and suboccipital trajectories has been predicated upon nuanced anatomical and pathological determinants. A pivotal inflection point in this narrative was reached in the 1980s, marking the apotheosis of microscopic neurosurgery as a globally acknowledged expertise. This monumental transition can be ascribed, in no small measure, to the erudite scholarship and pioneering endeavors of luminaries such as Malis and Yasargil. Their seminal expositions on the underlying dogmas and technical matrices of skull base surgery instigated a doctrinal metamorphosis, unequivocally enshrining microscopic neurosurgery as a cornerstone in the integrated therapeutic strategies for skull base pathologies [5].

## 2. Comprehensive Analysis of the Anterior, Middle, and Posterior Cranial Fossa

The anterior skull base constitutes a convex anatomical structure intricately composed of frontal, ethmoid, and sphenoid bones. Functioning as a partition, this thin osseous layer serves to segregate the intracranial contents from the sinonasal and orbital anatomical features. In terms of its specific anatomical constituents, the frontal bone forms the posterior wall of the frontal sinus and the roof of the orbit. The ethmoid bone, on the other hand, contributes to the architecture of the ethmoid sinus roof and the cribriform plate. Lastly, the planum sphenoidale and the anterior clinoid processes of the sphenoid bone establish the posterior component of the ASB. Vascular structures, specifically the posterior and anterior ethmoid arteries, demonstrate unique trajectories; the former typically courses almost directly from lateral to medial within the bone, whereas the latter exhibits more variability and may course obliquely from posterolateral to anteromedial. It is imperative to meticulously identify these vascular landmarks to avoid the potentially catastrophic complication of retrobulbar hemorrhage. Moreover, the cribriform plate features small bony channels that facilitate the passage of olfactory filae, accompanied by dural invaginations. This anatomical characteristic renders the region susceptible to both iatrogenic and spontaneous cerebrospinal fluid (CSF) leaks. The ethmoid roof, often being exceedingly thin, is also a frequent site for iatrogenic CSF leaks [6].

In a different anatomical context, dissection of the middle cerebral fossa (MCF) floor unveils a collection of crucial anatomical structures, which include the arcuate eminence, the greater superficial petrosal nerve (GSPN), the middle meningeal artery (MMA) and its corresponding foramen spinosum, the gasserian ganglion of the trigeminal nerve, the superior petrosal sinus (SPS), and the petrous internal carotid artery. To facilitate surgical dissection, three distinct anatomical landmarks within the MCF floor can be identified: Glasscock’s triangle, Kawase’s triangle, and Trautmann’s triangle. Glasscock’s triangle is demarcated by the foramen spinosum, V3 (the mandibular branch of the trigeminal nerve), and the groove for the GSPN, and is most notably associated with the location of the petrous internal carotid artery [7,8]. Kawase’s triangle, conversely, defines the region for bone removal medial to the internal carotid artery and is bordered by the gasserian ganglion, cochlea, GSPN, and carotid artery [9]. Trautmann’s triangle, located posterior to the internal auditory canal (IAC), is demarcated by the semicircular canals, the jugular bulb, and the adjacent posterior fossa dura in the vicinity of the sigmoid sinus and serves as a guide for posterior petrosectomy [10].

## 3. The Surgical Vanguard: Advanced Techniques and Modalities

### 3.1. Evolution and Optimization of Minimally Invasive Surgical Avenues

In the evolving landscape of neurosurgery, the initial adoption of endoscopy was notably slow-paced, a phenomenon that can be largely attributed to the concurrent rise and standardization of the surgical microscope within neurosurgical procedures. This process of standardization was not only effective but also influential enough to relegate the development of endoscopic methods to a lower priority within the scientific community [11].

This technological bifurcation manifested itself prominently in the surgical strategies targeting conditions related to the anterior skull base. Over the course of medical history, a panoply of surgical approaches—ranging from transcranial to transfacial methodologies—has been utilized, either as standalone techniques or in composite form. These procedures are often labeled as “aggressive” due to their invasiveness and complexity. They are frequently employed in oncological cases, particularly in patients whose overall health is already compromised. Interestingly, these aggressive methods have also been considered for the treatment of relatively benign conditions, such as cerebrospinal fluid fistula lesions [12].

However, the advent of Functional Endoscopic Sinus Surgery (FESS) marked a paradigmatic shift in the surgical management of anterior skull base pathologies. What commenced as an endoscopic technique primarily for diagnostic purposes eventually metamorphosed into an array of specialized surgical methodologies. Advances in endoscopic technology catalyzed the emergence of endoscopic endonasal approaches, providing surgeons with a more nuanced spectrum of options. These endoscopic techniques have been successfully adapted for a range of surgical applications, extending from transsphenoidal pituitary interventions to more elaborate endoscopic excisions involving the skull base [13,14].

Therefore, endoscopic methodologies have redefined the surgical portfolio, introducing minimally invasive options and thereby engendering a revolution in the treatment strategies for conditions involving the anterior skull base. The advancements in endoscopic technology offer a compelling alternative to traditional approaches, holding significant promise for both improving patient outcomes and broadening the scope of treatable conditions.

### 3.2. Innovations in Pituitary Surgery: A Confluence of Endocrinology and Neurosurgical Finesse

Pituitary adenomas, neuroendocrine tumors arising from the anterior pituitary gland, are traditionally classified as either functional or nonfunctional based on their endocrine secretory profiles. Among the functional adenomas, prolactinomas are most prevalent. For these tumors, dopamine-agonist pharmacotherapy serves as the cornerstone of treatment. Surgical intervention is generally considered a secondary option, typically reserved for those patients who exhibit pharmacological resistance despite dose escalation or who cannot tolerate medication-induced side effects. In contrast, adenomas that secrete adrenocorticotropic hormone (ACTH), leading to Cushing’s disease, or growth hormone, resulting in acromegaly, are predominantly managed with transsphenoidal surgical resection. The rates of biochemical remission postsurgery are substantially influenced by factors such as tumor size and the degree of invasiveness [15].

**Figure 1:**
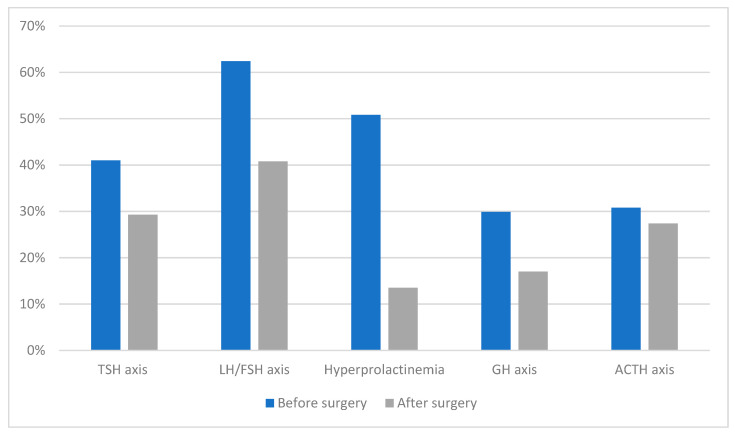
Endocrinological preoperative status compared to postoperative outcome, significant improvement of all pituitary hormones levels is shown.

Postoperative endocrinological outcomes (Figure 1) following transsphenoidal surgery for non-functioning pituitary macroadenomas (NFPAs) present an intricate landscape. Interestingly, the incidence of at least one new hormonal deficiency postoperatively is lower compared to the rate of recovery for at least one preexisting hormonal axis. Among the hormone secretory reserves, ACTH appears to be the most susceptible to postoperative deficit, while the thyroid-stimulating hormone (TSH) secretory reserve is relatively resilient. Gender differences also come into play, with men exhibiting a higher likelihood of recovery from preexisting central hormonal deficiencies subsequent to surgical intervention. Furthermore, the presence of hyperprolactinemia emerges as the most potent predictor for the restoration of pituitary function. These postoperative outcomes underscore the viability of surgical intervention for hypopituitarism resulting from NFPAs, owing to the promising rates of functional recovery and the relatively modest risk of inducing new hormonal deficiencies [16].

The therapeutic approach to pituitary adenomas is guided by the hormonal activity of the tumor, among other factors. For prolactin-secreting tumors, dopamine-agonist therapy is the primary treatment choice, while adrenocorticotropic hormone and growth hormone-secreting adenomas are often managed surgically via a transsphenoidal approach. Surgical options also hold promise for non-functional pituitary adenomas, particularly given the favorable postoperative hormonal recovery rates and low incidence of new hormonal deficiencies. This tailored approach, influenced by tumor functionality and patient-specific variables, represents the current state-of-the-art in pituitary adenoma management.

Recent meta-analyses have shed further light on the efficacy of endoscopic surgical techniques for pituitary adenomas. One such meta-analysis by Tabaee and colleagues [17] reported a gross tumor resection rate of 78% in a cohort of 821 patients. The hormonal remission rates for various adenoma types following endoscopic resection were either comparable to or surpassed those achieved through microsurgical techniques. A subsequent meta-analysis by Doward [18] included additional studies and reaffirmed these results, suggesting higher hormonal remission rates for functional microadenomas treated with endoscopic techniques (84%) compared to microsurgery (77%). This differential was even more significant for macroadenomas, with endoscopic techniques achieving a 70% remission rate versus 45% for microsurgical approaches.

The enhanced panoramic visualization and illumination provided by pure endoscopic techniques offer several advantages over traditional microsurgical approaches. These include the ability to operate in areas previously deemed challenging to access, such as the cavernous sinus, suprasellar region, planum sphenoidale, olfactory groove, and retroclival lesions [19]. These endoscopic advances not only increase the surgical field but also minimize nasal mucosal trauma, thereby reducing the need for nasal packing and postoperative discomfort unless significant cerebrospinal fluid leaks or mucosal bleeding occur [20]. 

Based on the existing literature, it is posited that the methodology employed by Bircher in accessing the cavernous sinus was pioneering in nature. Executed in 1892, this avant-garde approach was undertaken in response to a clinical presentation of thrombophlebitis in a female patient [21]. Moreover, an analytical endeavor aimed to understand the microsurgical and endoscopic anatomy of the cavernous sinus (CS), sixteen cadaveric craniums underwent detailed anatomical dissections. Of these, six were designated for transcranial evaluations, wherein three had their supratentorial cerebral contents excised for enhanced access to the CS and its contiguous structures [22]. Conversely, the remaining three retained their brains in situ during the study. Simultaneously, another subset of six specimens underwent endoscopic examination of the CS. Interestingly, four specimens were subjected to dual analyses—both transcranial and endoscopic—for parallel observations. Post-dissection assessments revealed that, while the CS and its pertinent anatomical structures were meticulously delineated in all craniums—ten transcranially and ten endoscopically—insights into their interrelationships were also garnered. It was discerned that, though cadaveric models afford uncomplicated microscopic and endoscopic visualizations of the CS, live surgical interventions within the CS present considerable challenges necessitating advanced surgical dexterity. Such cadaveric explorations underscore the feasibility of tailored approaches, be it transcranial microsurgery, endonasal endoscopy, or a symbiotic combination, contingent upon the specific pathological presentation and its spatial attributes [22].

In summary, endoscopic techniques in the surgical management of pituitary adenomas offer several advantages, including higher rates of tumor resection and hormonal remission, especially for challenging adenomas such as macroadenomas. The utility of these techniques, coupled with reduced postoperative morbidity, suggests a shifting paradigm in the surgical management of pituitary adenomas, offering both patients and clinicians more effective treatment options.

### 3.3. Cranial Base Reconstruction: A Mosaic of Biomaterial Science, Engineering, and Surgical Craftsmanship

The primary objective of skull base reconstruction is to create a durable, watertight barrier between the intradural contents and the external environment. This priority stems from the severe complications that can arise from persistent cerebrospinal fluid fistula, including meningitis and pneumocephalus, both of which can increase mortality over time [23]. Secondary goals include the closure of dead space, functional restoration, and aesthetic improvement. Various methods have evolved for reconstructing post-craniectomy defects using both synthetic and natural materials, aimed at both mitigating postoperative complications and enhancing cosmetic outcomes [22,23].

While open skull base surgery has traditionally been the standard of care for ablative margin control and definitive reconstruction, endoscopic skull base surgery has witnessed significant growth in popularity and is swiftly becoming the new standard in many centers. However, open approaches remain indispensable for certain complex conditions such as specific malignant tumors, larger composite defects, significant craniofacial trauma, osteoradionecrosis, and failed prior endoscopic reconstruction [24,25,26].

The choice of reconstructive approach often depends on the specific anatomical considerations. Anterior defects with minor dural damage or an intact bony ledge might do well with simpler reconstruction methods such as multilayered acellular alloplastic materials and free grafts, benefitting from the weight of the anterior intracranial contents to help seal underlay grafts or flaps [27]. Conversely, large posterior defects involving extensive bone and dural damage are usually more challenging to manage. These often necessitate the use of robust vascularized tissue and meticulous postoperative CSF pressure management, sometimes requiring permanent or temporary CSF diversion [28].

Additionally, the extent of the defect—including volume, loss of bony buttresses, and the presence of high-flow CSF leaks—must be thoroughly evaluated to tailor the surgical approach. In summary, both open and endoscopic techniques have distinct advantages and limitations, and the choice between them is guided by the specific needs of the case, taking into account factors such as the size and location of the defect, the risk of complications, and aesthetic considerations.

Various materials, both autologous and synthetic, have been employed in skull base reconstruction, serving different functional and anatomical needs. Autogenous grafts such as nasal mucoperichondrium and mucoperiosteum, tensor fascia lata, temporoparietal fascia, calvarial bone, and abdominal adipose tissue have all been documented for use in reconstruction [29]. On the synthetic front, noncellular materials such as DuraGen, AlloDerm, DuraSeal, and hydroxyapatite cements have been utilized [28,29].

For anterior skull base reconstructions, vascularized locoregional flaps have emerged as the go-to option. Specifically, the nasal septal flap, based on the posterior septal artery, has greatly advanced endoscopic skull base surgery. Its reliability, versatility, and low morbidity have made it the first-line choice in endoscopic reconstruction, significantly lowering the rates of CSF leaks [30,31].

Soft tissue donor sites for free flaps in skull base reconstruction have also diversified. The rectus abdominus is a well-established choice, known for its reliable deep inferior epigastric pedicle, as well as offering both large skin area and muscle bulk suitable for filling dead space [31,32]. Similar characteristics are found in the latissimus dorsi flap. Recently, the anterolateral thigh has become increasingly popular for its low morbidity, versatility, and long reliable vascular pedicle. Options such as the vastus lateralis muscle or vascularized tensor fascia lata can also be utilized either alone or in combination, adding to the arsenal of reconstructive choices [33,34,35].

The evolution of materials and techniques in skull base reconstruction has significantly broadened the surgical toolkit, offering a range of autogenous and synthetic materials for varying needs. These developments have contributed to more effective, reliable, and low-morbidity reconstructive options, enhancing both functional and cosmetic outcomes.

## 4. The Renaissance of “Functionally” Guided Surgery: Intraoperative Neuromonitoring

### 4.1. Historical Overview and Technological Breakthroughs

The history of Intraoperative Neuromonitoring (IONM) traces back to 1898, when Dr. Fedor Krause in Berlin used monopolar faradic stimulation during an acoustic nerve neurectomy. Krause’s work marked the earliest instance of visual observation of nerve activity during surgery. The technique gained significant momentum in the 1960s, when it was adapted for thyroid surgeries by Flisberg and Lindholm, and also for parotid and ear surgeries through facial nerve stimulators developed by Parsons and Hilger [36].

In contemporary medical practice, IONM has become a staple in various surgical disciplines, especially those involving close proximity to critical nerves. Predominant among these are thyroid surgeries—where the vagus and recurrent laryngeal nerves are monitored—parotidectomy, and surgeries of the posterior cranial fossa where facial nerve monitoring is crucial. During these operations, the surgeon employs a stimulator probe to accurately identify and differentiate the nerve from surrounding tissues. When the probe is placed onto the nerve, a circuit is closed that triggers either visual or auditory cues each time the nerve is contacted. This type of monitoring is often referred to as intermittent IONM (iIONM) [37].

IONM has evolved from its humble beginnings to become an integral part of modern surgery. It provides surgeons with real-time feedback, enhancing surgical precision and thereby potentially reducing post-operative complications. Its applications have been widely adopted in surgeries that risk nerve injury, making it a standard practice in many medical institutions.

### 4.2. Mechanisms, Modalities, and the Paradigm Shift towards Real-Time Functional Feedback

Brainstem Auditory Evoked Potentials (BAEPs) are bioelectric neural activities triggered by the stimulation of the vestibulocochlear nerve [38]. These potentials are particularly challenging to distinguish from the background electrical activity of the brain due to their relatively small amplitude [37,38]. To separate the BAEPs from this “noise”, thousands of samples of the electric stimulus are gathered and averaged, allowing for a clearer identification of the auditory evoked potential.

In BAEP recordings, data are collected from multiple points along the vestibular nerve pathway as it moves from the peripheral to the central nervous system [39,40]. The peaks in these recordings are categorized as Waves I through V, which correspond to different anatomical locations—from the peripheral cochlear nerve to the inferior colliculus [41,42].

During BAEP monitoring, electrodes are placed on the scalp and earlobes. An auditory stimulator then emits acoustic clicks to the ear being operated on, delivered through an earphone-transducer setup. The electrical pulse rate for these clicks is typically set between 20 to 50 per second. Before the operation starts, the stimulus intensity, usually measured in decibels, is adjusted to a level where the patient can hear the clicks. The final stimulus is then set at an intensity a few decibels higher than this initial threshold. To ensure focused monitoring, white noise is applied to the contralateral ear at a lower intensity to mask its response [41,42].

BAEPs are a critical tool for monitoring neural activity related to auditory functions during surgical procedures. They allow for real-time tracking of auditory pathway integrity, which is particularly useful in surgeries where the auditory nerve might be at risk. This method is complex and requires precise setup and interpretation, but its importance in safeguarding auditory function during surgical procedures is well recognized [43,44].

The monitoring of somatosensory spinal pathways, specifically the dorsal column-medial lemniscus, relies on subcortical and cortical responses to continuous electrical stimulation of peripheral nerves such as the tibial, peroneal, ulnar, or median nerve. This method of Intraoperative Neuromonitoring is commonly used and easy to implement, having no contraindications. It can be particularly useful for monitoring the posterior spine approach in spinal deformity surgeries, boasting a sensitivity range of 25–92% and a specificity of 96–100% [45]. However, it does have limitations, such as a time lag (1–20 min) in data interpretation due to signal averaging, making it possible for an injury to go undetected until it becomes irreversible. It is also less effective for monitoring patients with pre-existing neurologic deficits or in situations involving isolated motor pathway or nerve root injuries, which are better detected by Motor Evoked Potentials (MEPs) or Electromyography (EMG) [46].

MEPs are particularly sensitive for monitoring motor pathways in the anterior or central regions of the spinal cord and nerve roots. They serve as highly reliable indicators of corticospinal tract injuries and have proven especially useful for detecting spinal cord ischemia during spinal deformity correction [45]. This form of monitoring involves real-time, intermittent stimulation of the motor cortex and subsequent recording at muscles, preferably those rich in corticospinal tract innervations such as distal limb muscles. Transcranial stimulation can be either magnetic or, more commonly in surgical settings, electric (Transcranial Electric Motor Evoked Potentials or TceMEP). The electromyography signals, also known as Compound Motor Action Potentials (CMAP), are typically acquired through needle electrodes inserted bilaterally into the upper limbs. These serve as controls to differentiate systemic, anesthesia, and positioning-related changes [47].

Both somatosensory and motor evoked potentials offer valuable insights into neural integrity during spinal surgeries, albeit with distinct advantages and limitations. While somatosensory monitoring is generally easier to implement and can provide information about both sensory and motor pathways, MEPs offer real-time, direct monitoring of motor pathways, making them invaluable in surgeries where motor function is at high risk.

### 4.3. Neuromonitoring in Diverse Pathologies: Customized Approaches for Tailored Surgical Interventions

Direct stimulation of the facial nerve during posterior cranial fossa surgery has been explored by Amano, who used a ball-type electrode to stimulate the root exit zone of the facial nerve. This method was shown to be potentially useful for assessing the state of the facial nerve during surgery. By examining variables such as amplitude preservation ratio and the last maximal amplitude, the method could predict the likelihood of facial nerve palsy postoperatively according to the House–Brackmann (HB) grade [48].

Multipulse Transcranial Electric Stimulation (TES) provides another approach to continuous monitoring of the facial nerve. A cup electrode placed on the skull sends out clusters of electrical pulses that stimulate the corticobulbar pathway, allowing real-time monitoring of facial nerve function through facial nerve muscle motor evoked potentials (FNMEP). This method has been found to accurately predict the postoperative state of the facial nerve, with patients maintaining at least 50% of the baseline amplitude generally experiencing no more than mild deterioration in facial nerve function postoperatively [49].

In contrast to active continuous Intraoperative Neuromonitoring (acIONM), there are methods described as passive continuous IONM (pcIONM) that do not involve direct stimulation but rather analyze natural discharge patterns that occur during the surgical procedure. Free-running electromyography (EMG) is one such method used to monitor the facial nerve during neurosurgery. In this technique, patterns such as spikes, bursts, and trains in the EMG signal are analyzed to provide insights into nerve function. Prass and Lüders described different types of EMG signal patterns such as spikes, bursts, and trains, which they observed during posterior fossa surgeries on 30 patients [50].

Multiple methods exist for intraoperatively monitoring the facial nerve during posterior cranial fossa surgery. Each has its unique advantages and disadvantages. Direct stimulation offers the ability to assess the facial nerve’s function at specific times during the procedure, while continuous methods such as TES allow for ongoing, real-time monitoring. Passive methods such as free-running EMG offer a non-intrusive way to monitor the nerve by analyzing its natural activity during surgery.

Despite advances in Intraoperative Neuromonitoring, the retention rates for vestibulocochlear nerve function are not as favorable as those for the facial nerve. This discrepancy could arise from the intrinsic challenges of preserving auditory function, especially when dealing with large tumors and those that have extensive infiltration into the cerebellopontine angle [49,50,51].

In the context of spine surgery, the choice of monitoring modality depends on the approach used and the specific risks involved. For posterior approaches, somatosensory evoked potentials (SSEPs) may be sufficient. However, for anterior approaches, transcranial motor evoked potentials (MEPs) are typically recommended due to the risk of anterior spinal artery syndrome. Where nerve root or spinal cord deficits are a major concern, additional modalities such as spontaneous and triggered electromyography may be valuable. Multi-modal IONM is highly recommended for procedures such as spine deformity surgery or those involving intradural tumors [52,53]. Anesthesia should be adjusted to allow for the best possible IONM recordings, with specific anesthetic agents contraindicated for certain types of monitoring [54].

IONM is not just limited to spine or cranial surgeries. It is also used in a variety of other surgical fields such as vascular and cardiothoracic. Its utility extends to preventing perioperative peripheral nerve injury (PPNI), which could occur due to excess mechanical pressure and torsion on the limbs and neck during surgery [55].

The use of IONM is crucial for optimizing outcomes in various types of surgery. While it has shown great promise, there is still room for improvement, particularly in monitoring the vestibulocochlear nerve. The choice of monitoring technique should be tailored to the specific surgical approach and the associated risks, and multi-modal IONM is often recommended for complex cases.

## 5. The Digital Surgeon: Technological Synergies in Cranial Base Surgery

### 5.1. Endoscopy in the New Era: Advanced Imaging, Robotic Assistance, and Augmented Reality Overlays

Advancements in skull base surgery are increasingly leveraging the capabilities of virtual reality (VR) and augmented reality (AR). For instance, color-coded stereotactic VR models can be custom-tailored for individual surgical cases, providing a simulated operating field for surgeons and trainees [56]. These models offer invaluable opportunities for surgical education and preoperative simulations. Furthermore, VR technology can be integrated into real-time operative settings by overlaying 3D images onto microscopic or endoscopic views, thus enhancing spatial navigation capabilities for the surgeon [57].

AR technology appears to offer particular benefits to less experienced medical professionals. These systems serve not just as educational tools but also as potential substitutes for existing neural navigation technology. AR can offer both contextual information about underlying structures and direct patient perspectives, potentially revolutionizing conventional neural navigation systems [58].

Beyond surgery, AR also has applications in non-surgical and clinical management at the skull base. For example, it is used for ablating damaged nasal tissue and offers guidance on basic surgical plans and navigational protocols [59]. In cranio-maxillofacial procedures, AR plays a significant role in reconstructing cheekbones and offering data on the underlying structure, albeit without the capability for real-time modifications [60]. Many AR applications superimpose precollected, immersive data onto real endoscopic camera images. However, fields that lie outside the endoscopic view remain hidden to the medical team, necessitating further adaptations to fully realize the technology’s potential.

Moreover, the application of Augmented Reality in clinical settings, particularly in the management of base-of-the-skull pathologies, has been gaining significant attention in the medical community, as evidenced by multiple academic conferences exploring its potential [59,60]. A specific clinical model has been proposed, offering an extended observational perspective of the area under examination [61]. In this model, endoscopic images are displayed centrally, while the projection external to the endoscopic field of view is rendered virtually, utilizing pre-existing computerized tomography data. Such an integrated AR framework suggests that, following technological advancements and methodological refinements, AR applications may become increasingly prevalent across a broader spectrum of clinical scenarios necessitating heightened alertness and precision [62].

When it comes to the design of an ideal AR device for clinical applications, certain rigorous criteria must be met to ensure its functional efficacy and safety. The system should feature a focus marker and device alignment capabilities that are intuitive and minimally intrusive, particularly for the medical professional using it. Calibration adjustments should be undertaken before the initiation of the clinical procedure to minimize undue burden or cognitive load on the healthcare provider [63].

Furthermore, conventional imaging techniques that focus solely on two-dimensional visual data may suffer from limitations in perceived depth, thereby potentially compromising the practitioner’s situational awareness and decision making accuracy. To mitigate such limitations, it is advisable to incorporate depth cues to enhance the perceptual veracity of the rendered images [64]. Additionally, in applications where virtual 3D objects are superimposed onto endoscopic images, it becomes imperative to maintain parallax when the viewing position changes in order to preserve spatial relationships and depth perception.

In terms of data presentation, meticulous attention must be devoted to the structural layout of the AR interface. Inadequate design considerations can obscure critical information or induce visual discomfort, thereby diminishing the user experience and potentially compromising clinical outcomes. Therefore, it is essential to engage in an iterative design process, incorporating user feedback and empirical data, to optimize the AR interface and data presentation for the specialized needs of clinical practice.

### 5.2. Data-Driven Neurosurgery: Machine Learning, AI-Assisted Diagnosis, and Surgical Planning

The application of Radiomics in oncological diagnostics has emerged as a transformative approach in recent years, particularly in the preoperative assessment of various neoplastic conditions including prostate cancer, lung cancer, and an array of brain tumors such as gliomas, meningiomas, and brain metastases [63,64,65]. Traditional diagnostic methodologies that rely predominantly on qualitative assessments made by radiologists based on “visible” features, Radiomics facilitates the quantitative extraction of high-dimensional features as parametric data from radiographic images [66,67].

The incorporation of machine learning algorithms further enhances the analytical capabilities of Radiomics, offering unprecedented insights into the pathophysiological characteristics of lesions that are otherwise challenging to discern through conventional visual inspection [68,69]. Several studies have demonstrated the utility of Radiomics-based machine learning in the differential diagnosis of various brain tumors, thus indicating its prospective application in clinical decision making [70].

In the feature selection domain, Least Absolute Shrinkage and Selection Operator (LASSO) has been noted for its effectiveness in handling high-dimensional Radiomics data, particularly when the sample sizes are relatively limited [71,72]. LASSO distinguishes itself by its ability to avoid overfitting, making it an optimal choice for robust feature selection in Radiomics analyses.

Additionally, Linear Discriminant Analysis (LDA) serves as another valuable machine learning classification algorithm tailored for Radiomics applications. LDA seeks to identify and delineate boundaries around clusters belonging to distinct classes and projects these statistical entities into a lower-dimensional space to maximize class discriminatory power. Notably, it has been reported to retain substantial class discrimination information while reducing dimensionality [73,74,75].

Radiomics has extended its utility beyond diagnostic applications to prognostic evaluations, as exemplified in its role in both the diagnosis and treatment control rate prediction for chordoma [76]. Chordoma, a disease notorious for its refractory nature necessitating multiple surgical interventions and radiotherapeutic treatments, poses unique challenges for sustained disease control. In this context, Radiomic models built on features describing both the morphological shape and the genomic heterogeneity of the tumor have demonstrated superior performance in predicting the effectiveness of radiotherapy for tumor control. Such predictive capabilities underscore the potential benefits of Radiomics in enabling more targeted, efficient treatment regimens for diseases such as chordoma, thereby potentially reducing the need for repetitive, invasive procedures.

In another application, Radiomics-based machine learning algorithms have been shown to assist significantly in the preoperative differential diagnosis between germinoma and choroid plexus papilloma [77]. These two types of primary intracranial tumors often present with overlapping clinical manifestations and radiological features, yet they require markedly different treatment modalities. In addressing this diagnostic conundrum, high-performance prediction models have been developed using sophisticated feature selection methodologies and classifiers. These models suggest that Radiomics can offer a non-invasive diagnostic strategy with substantial reliability. 

Notably, the application of Radiomics and machine learning in these scenarios holds the promise of revolutionizing the approach to image-based diagnosis and personalized clinical decision making. By leveraging advanced computational techniques to analyze complex, high-dimensional radiographic data, Radiomics provides a more nuanced understanding of tumor characteristics and treatment responses. This computational approach thereby opens avenues for more accurate, timely, and individualized therapeutic strategies, significantly enhancing the quality of patient care in oncological settings.

**Figure 2:**
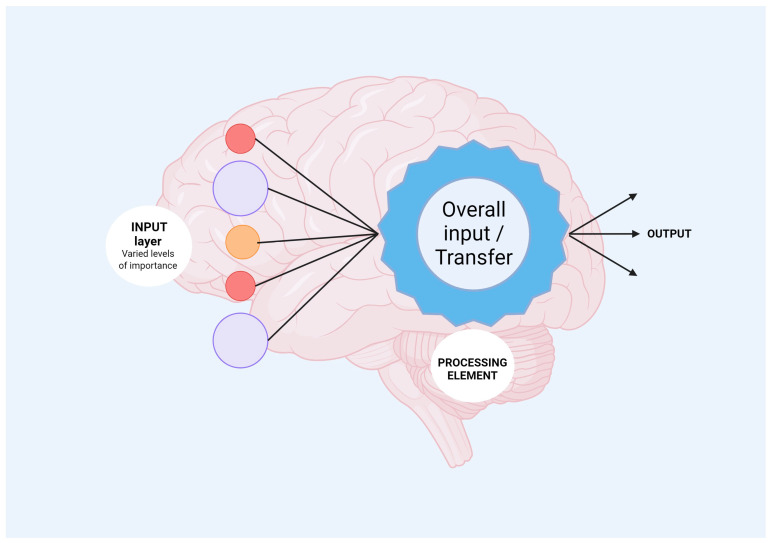
Mechanisms of neural network processing are shown. Input layer refers to heterogenous data which will be analyzed by the neural network incorporated algorithms. Further, output information is obtained, offering new avenues for biomedical fields.

In the realm of skull base neurosurgery, machine learning (ML) methods, including neural network models (NNs) (Figure 2), have been rigorously applied to a comprehensive, multi-center, prospective database to predict the occurrence of Cerebrospinal Fluid Rhinorrhoea (CSFR) following endonasal surgical procedures [78]. The predictive capabilities of NNs surpass those of traditional statistical models and other ML techniques in accurately forecasting CSFR events. Notably, NNs have also revealed intricate relationships between specific risk factors and surgical repair techniques that influence CSFR, relationships that remained elusive when examined through conventional statistical approaches. As these predictive models continue to evolve through the integration of more extensive and granular datasets, refined NN architectures, and external validation processes, they hold the promise of significantly impacting future surgical decision making. Such next-generation models may provide invaluable support for more personalized patient counseling and tailored treatment plans.

Regarding automated image segmentation in surgical navigation applications, although there is a high correlation between the automated segmentation and the anatomical landmarks in question, the Dice Coefficient (DC)—a measure commonly used to assess the performance of the segmentation task—was not deemed to be particularly high [79]. Various factors contribute to this finding, including the complexity of anatomical pathways, the absence of clearly delineated contours in certain regions, and inherent variations arising from manual segmentation. These limitations cast doubt on the utility of the DC as a standalone metric for objectively evaluating the performance of this specific task. However, the low average Hausdorff Distance (HD) on the testing dataset better encapsulates the high accuracy of the automated segmentation, bolstering its credibility for applications such as surgical navigation.

In summary, the application of machine learning, and particularly neural networks, appears to be a game-changer in predicting complex clinical outcomes such as CSFR following skull base neurosurgery. Meanwhile, automated image segmentation remains a challenging task, warranting a more nuanced approach to performance assessment than merely relying on singular statistical measures such as the Dice Coefficient. These advancements signify not only the growing impact of computational methods in medicine but also the necessity for ongoing refinement and validation to ensure these techniques meet the highest standards of clinical efficacy and safety.

## 6. Radiosurgery and Radiotherapy: Harmonizing Precision and Efficacy

### 6.1. An In-Depth Exploration of Radiosurgical Modalities: Gamma Knife, CyberKnife, and Beyond

Stereotactic radiosurgery has emerged as a pivotal treatment modality for various types of lateral skull base lesions, with perhaps its most significant impact being on the management of glomus jugulare tumors [80]. Many medical centers have adopted this approach as the first-line treatment for growing symptomatic tumors due to its lower morbidity compared to traditional surgical interventions, coupled with comparable rates of disease control.

Additionally, the efficacy of radiosurgery in treating skull base meningiomas has been well documented, with long-term follow-up data indicating impressive tumor growth control rates ranging from 92 to 98% [81]. While determining the optimal radiation dosage is critical, findings suggest that doses greater than 12 Gy to the tumor margin are essential for effective control. Suboptimal doses, specifically less than 12 Gy, have been associated with a significant tumor growth rate during one-year follow-up periods [82]. It is generally recommended that the minimum effective radiation dose for skull base meningiomas should be between 13 and 17 Gy, although the suitability of lower doses remains a topic of ongoing debate [83].

Importantly, some nuances exist within the treatment paradigm. For instance, Lee et al. noted that previously resected tumors might pose challenges in accurate radiological delineation due to postoperative changes such as meningeal enhancements or fat signals, which could be mistaken for tumor tissue [83]. Moreover, Zachenhofer and colleagues posited that tumor regrowth often occurs outside the targeted radiosurgical volume, a phenomenon possibly attributable to microscopic remnants of tumor cells within the adjacent dura mater that are not included in the radiosurgical target [84].

Stereotactic radiosurgery offers a promising avenue for managing various types of skull base tumors, including glomus jugulare and meningiomas, with both short-term and long-term efficacy. However, it is crucial to consider factors such as optimal radiation dosage and potential challenges related to the radiological delineation of previously resected tumors. These complexities underscore the necessity for personalized treatment strategies and underscore the importance of ongoing research to fine tune radiosurgical approaches for maximum clinical benefit.

The management of benign meningiomas using radiosurgery must be approached with caution given the potential for malignant transformation. up to 2% of benign meningiomas could transform into malignant forms, and others have found that 28.5% of recurrent benign meningiomas were actually atypical or anaplastic [83,84]. These statistics underline the importance of long-term monitoring and potential re-evaluation of treatment plans [85,86].

Radiosurgical interventions also present challenges related to cranial nerve sensitivity, most notably the optic and trigeminal nerves. The optic nerve is particularly vulnerable to radiation, requiring careful dose planning. Leber et al. suggested that doses below 10 Gy could be safely administered to the optic nerve without complications, but doses between 10–15 Gy carry a 26.5% risk of optic neuropathy [87]. Thus, tumors close to or compressing the optic apparatus are less amenable to radiosurgical treatment, as delivering an effective dose could jeopardize optic nerve function.

Various guidelines have been proposed for dosing the optic apparatus, with Morita et al. allowing for short segments to receive between 12–16 Gy [88], and Stafford et al. reporting no optic neuropathy with a 12 Gy dose [89]. Therefore, radiosurgery might be more appropriately indicated for tumors situated at least 5 mm away from the chiasm and optic nerve.

The trigeminal nerve also shows significant sensitivity to radiation, with various studies reporting the development of trigeminal neuropathy post-treatment. For example, Lee et al. found that 4% of their patients developed this condition, with a portion experiencing permanent deficits [83]. Moreover, Chang et al. reported that although 86% of patients initially experienced pain relief, about half suffered pain recurrence during the follow-up period [90]. Radiation doses exceeding 19 Gy were found to be associated with a high incidence of trigeminal neuropathy [88].

The utility and efficacy of radiosurgery for skull base meningiomas appear to be influenced by several factors including tumor size, dose, and fractionation. Single-session radiosurgery has been reported to yield a five-year actuarial tumor control rate of 88.6% for large skull base meningiomas (>8 cm^3^). However, tumor control rates tend to decrease with increasing tumor volume, specifically tumoral volumes ≥ 14 cm^3^ [91]. With a median dose of 10 Gy (ranging between 8–10 Gy), the five-year and ten-year tumor growth control rates were 78% and 70%, respectively. Notably, only 6% of patients experienced permanent radiation injury with an 84-month follow-up [92]. 

The CyberKnife^®^ platform offers a technological advancement in frameless robotic radiosurgery, enabling high precision and conformal intracranial tumor targeting. It allows for easy fractionation of treatment, thereby minimizing toxicity, especially when adjacent organs-at-risk (OAR) have low radiation tolerance [93]. However, the outcomes for larger, malignant tumors remain less predictable, with both tumor size and type affecting the treatment outcome [94].

In the case of smaller, radiosensitive tumors such as vestibular schwannomas and meningiomas, radiosurgery has been largely effective with minimal acute toxicity. However, areas for improvement include symptom management and late morbidity. The presence of larger tumors, less optimal dose/fractionation, and other risk factors such as previous cranial radiotherapy can lead to increased treatment-related toxicity [95].

Given these findings, the focus for smaller radiosensitive tumors should be on optimizing dose prescription and fractionation schedules. Careful planning that includes vigilance over multiple dose indices for susceptible OAR may help minimize late toxicity and optimize functional preservation. For tumors of other pathological types, which tend to be larger and/or more radioresistant, initial efforts should aim at increasing local control while minimizing toxicity through optimized dose and fractionation scheduling.

In summary, while radiosurgery has shown promising outcomes for skull base meningiomas and other cranial tumors, there are challenges that need to be addressed. Tumor size, type, and proximity to critical structures such as OAR can impact the efficacy and safety of treatment. Consequently, individualized treatment plans, leveraging advanced technologies such as CyberKnife^®^ and ongoing research, will be key to improving outcomes.

### 6.2. Radiotherapy Advancements: Modulating Doses, Fractions, and Protocols for Optimal Tumor Control and Preservation of Neural Structures

Fractionated stereotactic radiotherapy (FSRT) has emerged as another viable option for the treatment of large skull base meningiomas. Studies have shown that FSRT can offer five-year tumor growth control rates ranging between 93–96%. In terms of toxicity, late clinical toxicity has been reported to be relatively low, falling in the range of 1.6–5.5%. The treatment generally involves delivering radiation doses of 50–56.8 Gy for tumor volumes averaging between 35.4–52.5 cm^3^. The mean duration of follow-up in these studies was between 35–42 months [96].

In a more recent study that compared single-session gamma knife surgery (GKS) with fractionated GKS (FGKS) for meningiomas having a volume greater than 10 cm^3^, FGKS appeared to show a marginally higher overall five-year tumor control rate (92.9% for FGKS vs. 88.1% for single-session GKS). However, it is important to note that the difference in the control rates between the two groups was not statistically significant (*p* = 0.389). The mean tumor volume for the single-session GKS group was 15.2 cm^3^, while for the FGKS group, it was 21 cm^3^. The FGKS group also included 16 skull base meningiomas [97].

Fractionated radiation therapy, which involves daily treatments usually spanning several weeks, is a commonly employed strategy for treating certain types of tumors, including WHO grade I meningiomas that are located close to sensitive areas such as the optic chiasm or optic nerves [98]. This approach is backed by evidence showing that external beam radiation therapy (EBRT) can deliver effective doses that control the tumor while preserving visual function [99] (Table 1).

In cases where meningiomas affect the optic nerve sheath, EBRT is the treatment of choice. Many patients have reported vision improvement following this treatment. Remarkably, no other treatment modalities, including surgical interventions, have been shown to improve vision to the same extent as radiation therapy (RT) for this specific patient group. Therefore, surgical decompression is typically reserved for patients with intracranial extensions and rapidly deteriorating conditions [116].

When it comes to cavernous sinus and petroclival meningiomas, radiation therapy is often the preferred treatment option, either as a primary treatment or as an adjunct to subtotal resection. These locations are associated with a high risk of surgical morbidity if extensive resection is attempted. A recent literature review indicated that stereotactic radiosurgery (SRS) alone resulted in a relatively low recurrence risk of about 3%. In contrast, more invasive procedures such as subtotal resection (STR) and gross total resection (GTR) had recurrence risks of around 11%. Moreover, cranial nerve deficits were more commonly reported among patients who underwent surgical resection [112].

Although chordomas are generally slow-growing tumors, aggressive upfront management has shown significant benefits in long-term survival. A retrospective study conducted in France demonstrated that patients who received RT immediately following surgery had a 10-year survival rate of 65%, whereas none of the patients who only received RT at the time of recurrence survived up to 10 years [117]. In the largest series on chordomas treated with RT, conducted at Harvard University, patients were treated with 60 to 79.2 Cobalt-Gray-Equivalent (CGE), and the local control (LC) rates at 10 years were found to be 44% [118]. A recent review that aggregated data from over 400 patients found that 5-year LC rates were close to 70% and overall survival (OS) was more than 80% [119].

Soft tissue sarcomas of the skull base are usually approached with maximal surgical excision, followed by post-operative radiation therapy. Recurrence risk is higher in these cases compared to soft tissue sarcomas of the extremities, mainly because obtaining clean surgical margins is often challenging. Various radiation therapy techniques are utilized, including external beam radiation therapy (EBRT), stereotactic radiosurgery (SRS), intraoperative RT, and brachytherapy [120].

Modern advancements in EBRT include technologies such as three-dimensional conformal RT (3D-CRT) and intensity-modulated radiation therapy (IMRT). Three-dimensional conformal RT typically delivers radiation from multiple angles in a coplanar fashion, akin to the spokes on a wheel. IMRT, on the other hand, allows the intensity of radiation beams to vary at different positions. This has significantly improved the ability to treat tumors located near sensitive structures, thereby advancing the field of radiation oncology [120].

Advances in radiation therapy and aggressive upfront management strategies have shown promising results in the treatment of chordomas and soft tissue sarcomas of the skull base. These findings underscore the need for individualized, multidisciplinary treatment approaches to optimize long-term outcomes.

## 7. Holistic Approaches: Interdisciplinary Collaborations and Patient-Centric Care

### 7.1. The Ecosystem of Cranial Base Surgery: Integrating Neurology, Radiology, Oncology, and Rehabilitation

The complexities involved in the surgery of skull base meningiomas (SBMs) increasingly point to the need for a multimodal treatment approach, integrating both radiosurgery and radiation therapy. This combination aims to maximize both functional outcomes and tumor control. Advances in technology, genomics, and Radiomics are poised to greatly enhance our understanding of tumor biology. This, in turn, allows for the tailoring of treatment plans in line with the tenets of precision medicine [121].

Beside multiple implicated medical specialties, neurosurgeons need to undergo a continuous high standard training for skull base pathology. Achieving surgical proficiency is paramount for educators within the domain of skull base surgery. Diligent effort, coupled with consistent and immediate evaluative feedback, constitutes a cornerstone of successful skill acquisition. Establishing an environment rooted in patient-centric values that fosters scholastic excellence augments the efficacy of a training program. Moreover, the usage of 3D printed models of the skull are currently used as a training possibility even for during the residency program. In the case of skull base pathologies, neurosurgeons can exercise the surgical approaches, especially various types of craniotomies on those synthetic-based models. For optimal knowledge assimilation, it is imperative that both the mentor and mentee engage proactively and with deliberate intent [122,123,124].

Given the rapidly evolving landscape of SBM treatment—fueled by technological and scientific innovations—a specialized multidisciplinary approach has become essential for optimal patient care. This has led to the conceptualization of “Centers of Excellence”, institutions specifically geared towards SBM management. These centers are not only technologically advanced but also guarantee an adequate workload for healthcare providers, ensuring they remain at the forefront of the field.

Moreover, integration of a diverse array of medical and allied health disciplines has the potential to substantially augment the quality of healthcare delivery, particularly in the context of Skull Base multidisciplinary teams. In such specialized tertiary referral centers, the amalgamation of expertise from various subspecialties not only fosters a holistic approach to patient care but also enhances the precision and efficacy of diagnosis, treatment planning, and execution.

Within these multidisciplinary frameworks, palliative care physicians contribute to symptom management and quality-of-life improvement, offering critical perspectives on end-of-life care when required. Neurosurgical anesthetists bring a refined understanding of perioperative management, particularly vital in the intricate surgeries associated with skull base anomalies. Chronic pain specialists offer insights into long-term analgesic strategies, thus contributing to sustained patient comfort and improved functionality post-surgery.

Similarly, clinical psychologists can play a pivotal role in assessing and addressing the psychological comorbidities often accompanying chronic or severe medical conditions. They provide cognitive-behavioral interventions and other psychological supports to enhance patients’ coping mechanisms. Audiological scientists and hearing and/or balance therapists contribute expertise on auditory and vestibular systems, which are frequently involved in skull base pathologies. Their input can be invaluable in both the diagnostic and rehabilitative phases of care.

Additionally, maxillofacial prosthetists offer specialized interventions that focus on reconstructive options, including facial prosthetics, which can be instrumental in postoperative rehabilitation. Speech and language therapists address communication and swallowing challenges that might arise due to anatomical changes or neurological impairments associated with skull base disorders. Dietitians further enrich the multidisciplinary team by offering tailored nutritional plans, thereby optimizing patients’ metabolic states for improved outcomes in both surgical and nonsurgical interventions.

This expansive collaborative approach is further fortified by interdepartmental interactions with neurosurgeons, neuroradiologists, and neuropathologists. Neurosurgeons offer specialized surgical interventions, while neuroradiologists provide crucial imaging expertise, enhancing the specificity and sensitivity of diagnostic processes. Neuropathologists contribute by offering detailed tissue diagnoses, which are vital for optimal treatment planning.

Given the complex, multifaceted nature of the conditions encountered in skull base pathology, and the necessity for sophisticated diagnostic and therapeutic modalities, the persistence of skull base multidisciplinary teams as a feature of tertiary referral centers seems not only likely but also clinically imperative. This convergence of specialized skills in a collaborative environment serves to enhance patient outcomes, facilitating a more nuanced and comprehensive standard of care [125].

### 7.2. Patient Narratives and Quality of Life Metrics Post-Surgery

Patients ultimately want their surgical team to cure, control, or, ideally, facilitate the prevention of disease. They favor minimally invasive approaches. When possible, they want illnesses to be treated by medicines only. If further intervention is necessary, they prefer minimally invasive surgery or radiosurgery without tissue damage. When more extensive surgery cannot be avoided, they prefer it to be without undue risk. Patients rightly place a premium on minimizing morbidity, which means no damage to the surrounding brain, cranial nerves, or blood vessels and no cosmetic deformity. Regardless of the approach, they want to minimize time away from work and family and to be treated at a reasonable cost [126].

## 8. Conclusions—Epilogue: Gazing into the Future Horizon

### 8.1. Challenges, Opportunities, and the Trajectory of Cranial Base Surgery in the Coming Decade

The adoption of 3D printing technologies is on the rise across various sectors, including neurosurgery. Current applications in this field encompass the fabrication of cranioplasty implants, educational models for tumors and aneurysms, as well as surgical planning aids [127]. Further innovation comes from the Northwestern University School of Engineering, where researchers have developed 3D-printed, patient-specific bioresorbable intravascular stents. Notably, a proof-of-concept for a 3D-printed bionic ear has been developed, featuring advanced auditory sensing capabilities for radiofrequency signals [128,129].

In the realm of cranial surgery, various robotic technologies are making headway. The NeuroArm, developed at the University of Calgary, is a remote-controlled surgical robot designed for use in an MRI suite [130]. Meanwhile, the Shinshu University NeuRobot—a joint effort involving multiple research institutions—consists of a master–slave micromanipulator system equipped with a rigid endoscope and three robotic arms, designed for minimally invasive procedures. This system has already been successfully employed in surgeries and shows potential for remote telesurgical applications, albeit with a minuscule 1 ms delay [131].

Currently, a team at the University of Washington is developing an Artificially Intelligent Neurosurgical Robotic Assistant. This autonomous robot aims to replicate the functions of a microneurosurgical assistant, such as gentle tissue manipulation and precise suction within the surgical field.

The development of an Artificially Intelligent Neurosurgical Robotic Assistant aims to create an intuitive system that can act according to the surgeon’s needs, either through innate understanding or voice commands. One of the major challenges lies in understanding the nuanced interaction between the surgeon and their assistant during surgery. To this end, the team has employed convolutional neural networks to analyze the surgeon’s voice and tool movements captured under a microscope [132]. Python speech application program interfaces are also used for more detailed analysis.

Instrument identification and tracking in the surgical field are performed at the pixel level, offering insights into the surgeon’s intended direction of movement [133]. An integral part of the project involves adapting natural language parsing to recognize specific medical terms, making the interface between the robotic assistant and the surgeon more intuitive and efficient.

In a novel application, the team recently showcased the fusion of semi-autonomous robotic therapy with a specialized biomarker known as “tumor paint”, derived from a component of scorpion toxin [134]. This biomarker specifically labels brain tumors. In studies led by Hu et al., a robotic system scanned a simulated tumor margin for spots marked as positive for tumor cells. Following the surgeon’s approval, the robot then executed an automated ablation pathway to remove these areas [135].

In summary, the convergence of robotics, machine learning, and specialized biomarkers is pushing the boundaries of what is possible in neurosurgical interventions. The fusion of these technologies could revolutionize how surgical procedures are planned and executed, with the promise of more precise and potentially less invasive treatments (Table 2).

### 8.2. Potential Breakthroughs: Stem Cell Research, Regenerative Medicine, and Genomic Tailoring

Stem cell recovery techniques are poised to play a transformative role in the treatment of surgically induced and other neurological deficits within the next two decades. These advancements could especially benefit patients requiring surgery for conditions such as vestibular schwannoma, promising better recovery of cranial nerve 7 and 8 function even for those with large or giant tumors. This promising approach could also extend to iatrogenic neurological deficits that may arise after surgeries on the brain or brainstem for tumor or vascular operations. Furthermore, understanding how stem cells interact with tumors may pave the way for the prevention and potential cure of some skull base malignant neoplasms [126]. 

In a notable development, a 12-month phase II, randomized controlled trial conducted in the US and Japanese centers showed that SB623 stem cells were particularly effective for patients with traumatic brain injury. These cells were implanted around the injury site, leading to significant improvements in motor function as measured by the Fugl-Meyer motor scale. The primary endpoint was reached, with an average improvement of 8.3 points as opposed to an improvement of 2.3 in the control group at 24 weeks (*p* = 0.040). This promising result has led SanBio Co., Ltd. (Tokyo, Japan), to plan further studies in phase III clinical trials, as per a personal communication from Steinberg GK in 2020 and a press release from SanBio Co., Ltd., in 2019 [136]. 

Like any groundbreaking medical advancement, the journey of integrating stem cells into clinical practice will require significant financial investment and time. It is also important to brace for setbacks and challenges along the way, much like the development pathways for new drugs or vaccines. Nevertheless, the prospects are exciting and could herald a new era in the treatment of neurological conditions and deficits.

### 8.3. Reiterating the Ethos of Continuous Learning, Global Collaboration, and Patient-First Principles

The future of skull base surgery and neurosurgery will undeniably be influenced by rapid technological advancements. While surgeons will need to be agile in integrating these new technologies into their practice, the core tenets that define a great surgeon—knowledge, innovation, technical skill, judgment, and compassion—will stand the test of time. Active engagement with emerging technologies is not just an option but a necessity, as it allows surgeons to have a direct hand in shaping the future of their field [126].

Innovation will be a linchpin in the evolution of medical practice, both now and in the future. These innovations might be subtle, influencing the minutiae of day-to-day work, or they could be groundbreaking, transforming clinical surgery, basic neurosciences, or various aspects of healthcare delivery. They might also aim at improving workflow and efficiency, revamping outpatient and hospital infrastructure, elevating patient satisfaction, or enhancing quality metrics [126]. 

Young surgeons carry the mantle of responsibility to not only excel in their craft but also to contribute to its progression. They must constantly aspire to leave their field better than how they found it, pushing the boundaries of what is possible and effective in medical care. Furthermore, surgeons should not shy away from roles in hospital and healthcare administration. Such involvement provides them with the opportunity to guide transformative changes, ensuring that innovation and quality improvement are not just theoretical ideals but real-world practices that enhance patient care and outcomes.

## Figures and Tables

**Table 1:** Pertinent studies on the use of additional radiotherapy in managing WHO grade II and III meningiomas.

Studies	Treatments	Histology	Results	Reference
Aghi et al., 2009	Surgery (TR), surgery + EBRT	WHO II	Relapse rates at 5 years were 41% and it was reduced to 0% with the inclusion of EBRT.	[100]
Attia et al., 2012	SRS	WHO II	TS exceeds 50% after 5 years; A 5-year LRR of 44%	[101]
Goyal et al., 2000	Surgery, surgery + FRT	WHO II	TR = 5-year TS was 87% PTR = 5 year TS was 100%	[102]
Huffman et al., 2005	GKRS	WHO II	40% relapse between 18 and 36 months.	[103]
Dziuk et al.,1998	Primarily surgery + FRT	WHO III	The 5-year TS is 57%.	[104]
Goldsmith et al., 1994	Surgery + FRT	WHO III	58%	[105]
Rosenberg and Prayson et al., 2009	Surgery + FRT; Surgery + SRS.	WHO III	5-year TS = 47%	[106]
Mattozo et al., 2007	SRS + EBRT	WHO I–III	Grade II: 3-year RFS rate of 83% Grade III: 3-year RFS rate of 0%	[107]
Adeberg et al., 2012	EBRT, surgery + EBRT	WHO II–III	Grade II tumors: 5-year TS rate of 81% and a 5-year RFS rate of 50%. Grade III tumors: 5-year TS of 53% and a RFS rate of 13%.	[108]
Hug et al., 2000	EBRT + surgery	WHO II–III	Grade II: 5-year TS rate of 38% Grade III: 5-year TS rate of 52%	[109]
Milosevic et al., 1996	Mainly surgery + FRT	WHO II–III	5-year TS rate of 28%	[110]
Pasquier et al., 2008	Surgery + EBRT, Surgery only	WHO II–III	TR: 5-year TS = 46% RT: 5-year TS = 0%	[111]
Sughrue et al., 2010	Surgery + FRT	WHO II–III	61%, 40% after 10 years	[112,113]
Yang et al., 2008 (33 atypical), (41 anaplastic)	Surgery, Surgery + EBRT	WHO II–III	Grade II: TS of 11.9 yrs and a RFS of 11.5 yrs (cases of atypical meningiomas) Grade III: TS of 3.3 yrs and RFS of 2.7 yrs	[114]
Boskos et al., 2009	EBRT Protons and photons	WHO II–III	5-year TS = 65% 5-year LRR = 61%	[115]

(TS = total survival, RFS = recurrence-free survival, EBRT = external beam radiation therapy, LRR = local recurrence rate, PTR = partial resection (STR), TR = total resection (GTR), FRT = Fractionated radiotherapy (RT), SRS = stereotactic therapy (radiosurgery), GKR = Gamma Knife radiosurgery).

**Table 2:** Future Advances in Various Fields in Skull Base Surgery.

Virtual Raman Microscopy and Spectroscopy for Quick Diagnosis in the Operating Room
Stem cell-based therapies for brain and cranial nerve damage from trauma, tumors, and medical procedures
Semi-autonomous robots for use in the operating room
Regenerative medicine combined with 3D printing for creating blood vessels, bone, and facial tissues
Quick molecular and genetic assessment of tumors
New training procedures for surgeons
Nanoengineering for diagnostic and therapeutic applications
Mobile imaging in the operating room and in the intensive care unit
CRISPR CAS-9 based genetic techniques to eliminate hereditary syndromes
Anti-cancer antibodies, CAR-T-cell therapy, and immune checkpoint blockade against the neoplasms
AI applications for powered disease diagnosis in hospital and outpatient care
Advanced imaging techniques (MRI and ultrasound)
Additive manufacturing (3D printing and rapid prototyping)

## Data Availability

All data is available online on libraries such as PubMed.

## Abbreviations

ASB	Anterior Skull Base
ACFR	Anterior craniofacial resection
CSF	Cerebrospinal fluid
GSPN	Greater superficial petrosal nerve
MCF	Middle cerebral fossa
IAC	Internal auditory canal
FESS	Functional Endoscopic Sinus Surgery
ACTH	Adrenocorticotropic hormone
NFPAs	Non-functioning pituitary macroadenomas
TSH	Thyroid-stimulating hormone
IONM	Intraoperative Neuromonitoring
iIONM	Intermittent IONM
BAEPs	Brainstem Auditory Evoked Potentials
MEPs	Motor Evoked Potentials
EMG	Electromyography
TceMEP	Transcranial Electric Motor Evoked Potentials
CMAP	Compound Motor Action Potentials
HB grade	House–Brackmann
TES	Transcranial Electric Stimulation
FNMEP	Facial nerve muscle motor evoked potentials
acIONM	Intraoperative Neuromonitoring
pcIONM	Passive continuous IONM
EMG	Electromyography
SSEPs	Somatosensory evoked potentials
MEPs	Motor evoked potentials
PPNI	Perioperative peripheral nerve injury
VR	Virtual reality
AR	Augmented reality
LASSO	Least Absolute Shrinkage and Selection Operator
LDA	Linear Discriminant Analysis
ML	Machine learning
CSFR	Cerebrospinal Fluid Rhinorrhoea
NNs	Neural network models
DC	Dice Coefficient
HD	Hausdorff Distance
OAR	Organs-at-risk
FSRT	Fractionated stereotactic radiotherapy
GKS	Gamma knife surgery
FGKS	Fractionated GKS
EBRT	External beam radiation therapy
TS	Total survival
RFS	Recurrence-free survival
EBRT	External beam radiation therapy
LRR	Local recurrence rate
PTR	Partial resection
TR	Total resection
FRT	Fractionated radiotherapy
SRS	Stereotactic therapy (radiosurgery)
GKR	Gamma Knife radiosurgery
RT	Radiation therapy
SRS	Stereotactic radiosurgery
STR	Subtotal resection
GTR	Gross total resection
CGE	Cobalt-Gray-Equivalent
LC	Local control
OS	Overall survival
EBRT	External beam radiation therapy
SRS	Stereotactic radiosurgery
3D-CRT	Three-dimensional conformal RT
IMRT	Intensity-modulated radiation therapy
SBMs	Skull base meningiomas
